# High resolution CT and histological findings in idiopathic pleuroparenchymal fibroelastosis: Features and differential diagnosis

**DOI:** 10.1186/1465-9921-12-111

**Published:** 2011-08-23

**Authors:** Sara Piciucchi, Sara Tomassetti, Gianluca Casoni, Nicola Sverzellati, Angelo Carloni, Alessandra Dubini, Giampaolo Gavelli, Alberto Cavazza, Marco Chilosi, Venerino Poletti

**Affiliations:** 1 Department of Radiology, Scientific Institute for study and treatment of Cancer-IRST; Via Piero Maroncelli 40-42; 47014; Meldola-Forlì; Italy; 2 Department of Pulmonology; Morgagni-Pierantoni Hospital; Via Carlo Forlanini 34, 47121; Forlì; Italy; 3 Department of Radiology; University of Parma; Via Gramsci 14; 43100; Parma, Italy; 4 Department of Radiology; Santa Maria Hospital; Via Tristano di Joannuccio 1, 05100; Terni, Italy; 5 Department of Pathology; Morgagni-Pierantoni Hospital; Via Carlo Forlanini 34, 47121; Forlì; Italy; 6 Department of Pathology; Arcispedale Santa Maria Nuova; Viale Risorgimento 57; 42100; Reggio Emilia; Italy; 7 Department of Pathology; University of Verona; Piazzale A. Stefani 1; 37126; Verona; Italy

## Abstract

Idiopathic pleuroparenchymal fibroelastosis (IPPFE) is a recently described clinical-pathologic entity characterized by pleural and subpleural parenchymal fibrosis, mainly in the upper lobes. As this disease is extremely rare (only 7 cases have been described in the literature to date) poorly defined cases of IPPFE can go unrecognized.

The clinical course of disease is progressive and prognosis is poor, with no therapeutic options other than lung transplantation currently available, yet. The aim of this report is to describe two further cases of this rare disease, reviewing CT, clinical and histological features.

## Letter

Idiopathic pleuroparenchymal fibroelastosis (IPPFE) is an entity characterized by pleural and subpleural parenchymal fibrosis. It is extremely rare (only 7 cases have been described in the literature to date) and was first described in 2004 by the Interstitial Lung Disease Program of the National Jewish Medical and Research Center of Denver [1].

Actually between 1996 and 2001, 5 cases were registered as cryptogenic syndrome with significant chest symptoms, radiographic pleura-parenchymal abnormalities and fibroelastotic changes seen on surgical biopsy specimens, without any evidence of other connective tissue disease [1]. Marked apical pleural thickening associated with superior hilar retraction is present at chest X ray analysis, and High Resolution Computed Tomography (HRCT) shows pleural thickening, fibrosis, architectural distortion, traction bronchiectasis and honeycomb lung [2]. The clinical course of this affection is progressive and prognosis is poor, with no therapeutic options other than lung transplantation available.

We here describe two additional cases of this rare disease.

**Figure 1:**
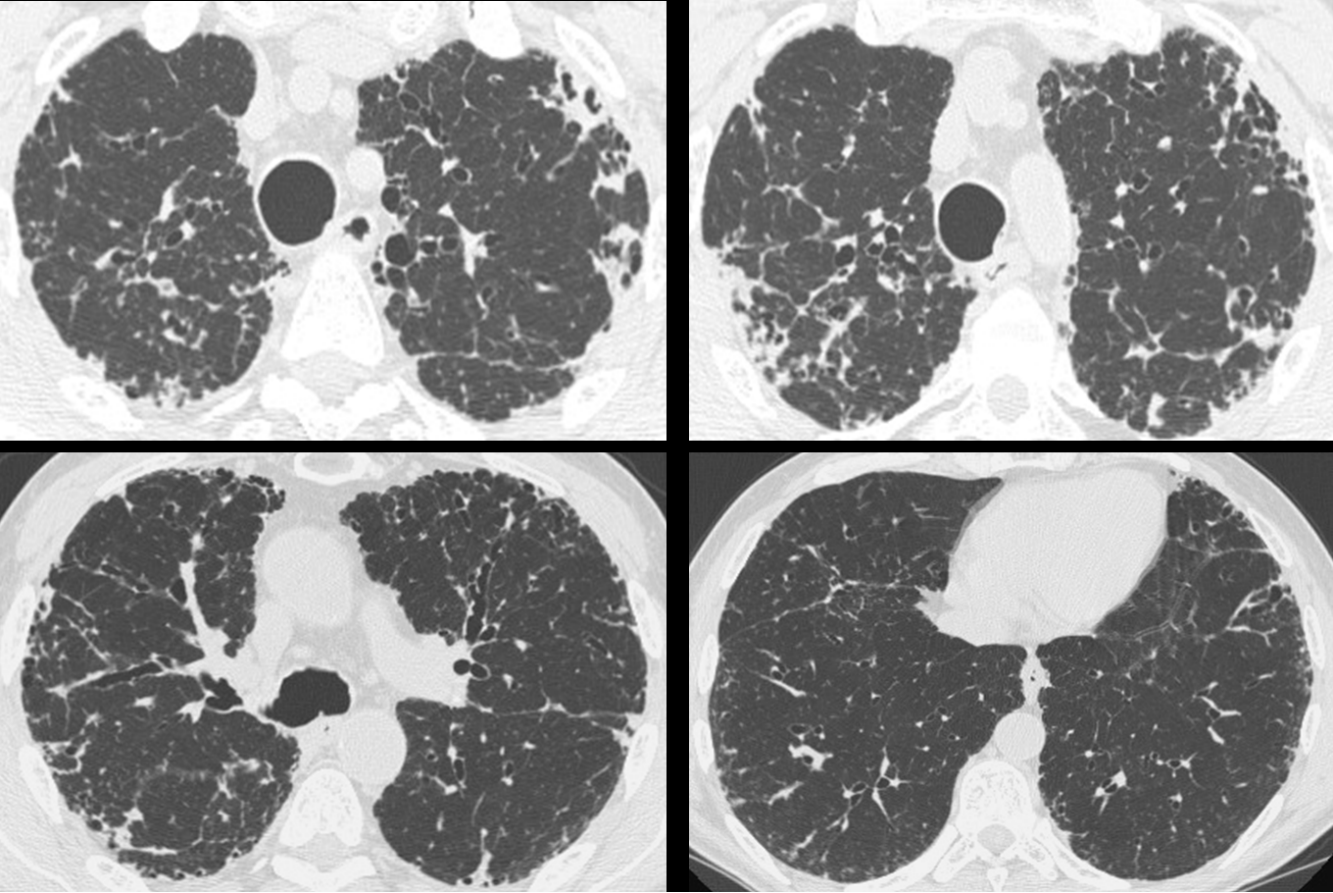
**High resolution CT scan in patient no. 1 shows severe pleural and subpleural thickening with moderate fibrotic changes in the marginal parenchyma**. Traction bronchiectasis and honeycombing are also seen.

**Figure 2:**
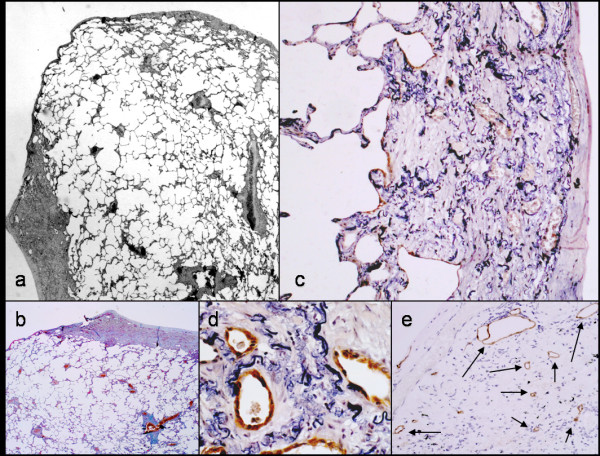
**Histological features of patient no. 1**. Marked subpleural thickening characterized by abnormal increase of elastic fibers and abrupt transition to normal parenchyma is evident at low magnification (2A and B). A number of residual alveolar structures are entrapped within the thickened pleural wall together with numerous lymphatic vessels (2c-e, podoplanin immunohistochemistry).

A sixty-eight-year-old man non-smoker, ex-fishmonger, living in the  countryside, presented to our institution with a three years long non-productive cough. Nine years earlier, the patient had been treated for gastric cancer, with subsequent negative follow-up examinations. No clubbing or other respiratory signs were observed upon physical examination. Lung functional exams were as follows: FVC 2.74 L-78%; FEV1: 99% and DLco 67%. No O2-desaturation during the a 420 m walk test was observed and final pO2 saturation was  96mmHg. Serological examination did not identify any sign of collagen-vascular disorders. CT scan (Figure 1) showed bilateral fibrotic changes, mainly represented in the upper lobes. Interlobular septal thickening, mild honeycombing and moderate pleural thickening were also seen. CT scan was blindly evaluated by two chest radiologists. Localization of abnormalities mainly in the upper lobes concerned radiologists of a possible chronic hypersensitivity pneumonitis (HP). Differential diagnosis included a non-typical pattern of usual interstitial pneumonia (UIP). Patient underwent bronchoalveolar lavage which showed 11% lymphocytes and 2% neutrophils. No eosinophils were seen. A transbronchial biopsy was performed too, but unfortunately nondiagnostic. As radiological and histological patterns were not suggestive of idiopathic pulmonary fibrosis and did not meet American Thoracic Society criteria, an open  lung biopsie (Video-Assisted Thoracoscopic Surgery) were performed on the dorsal segments of the right upper and lower lobes. VATS showed  histological features suggestive of idiopathic pleuroparenchymal fibroelastosis (IPPFE) (Figure 2).  

**Figure 3:**
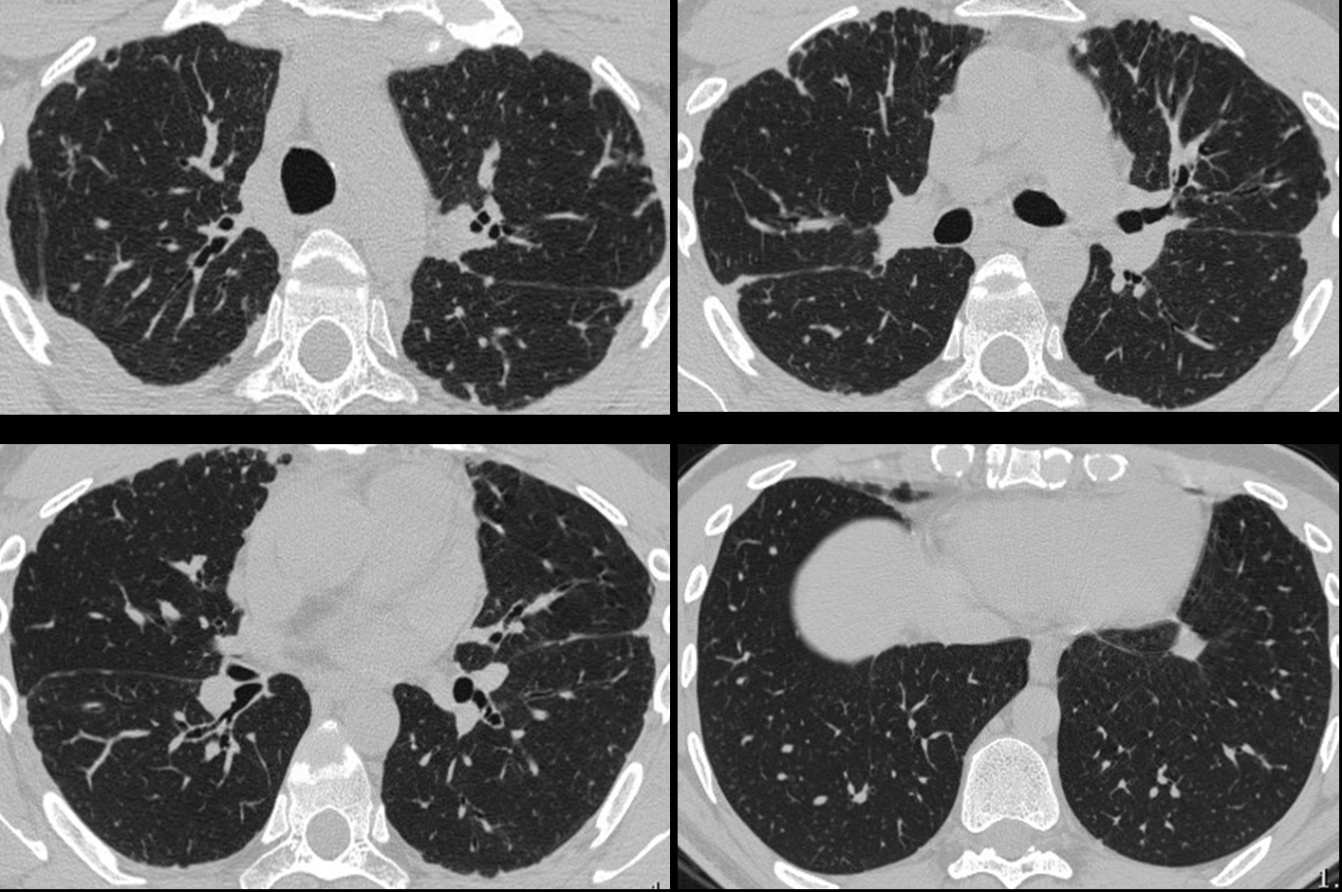
**High resolution CAT scan in patient no. 2 shows pleural thickening**.

**Figure 4:**
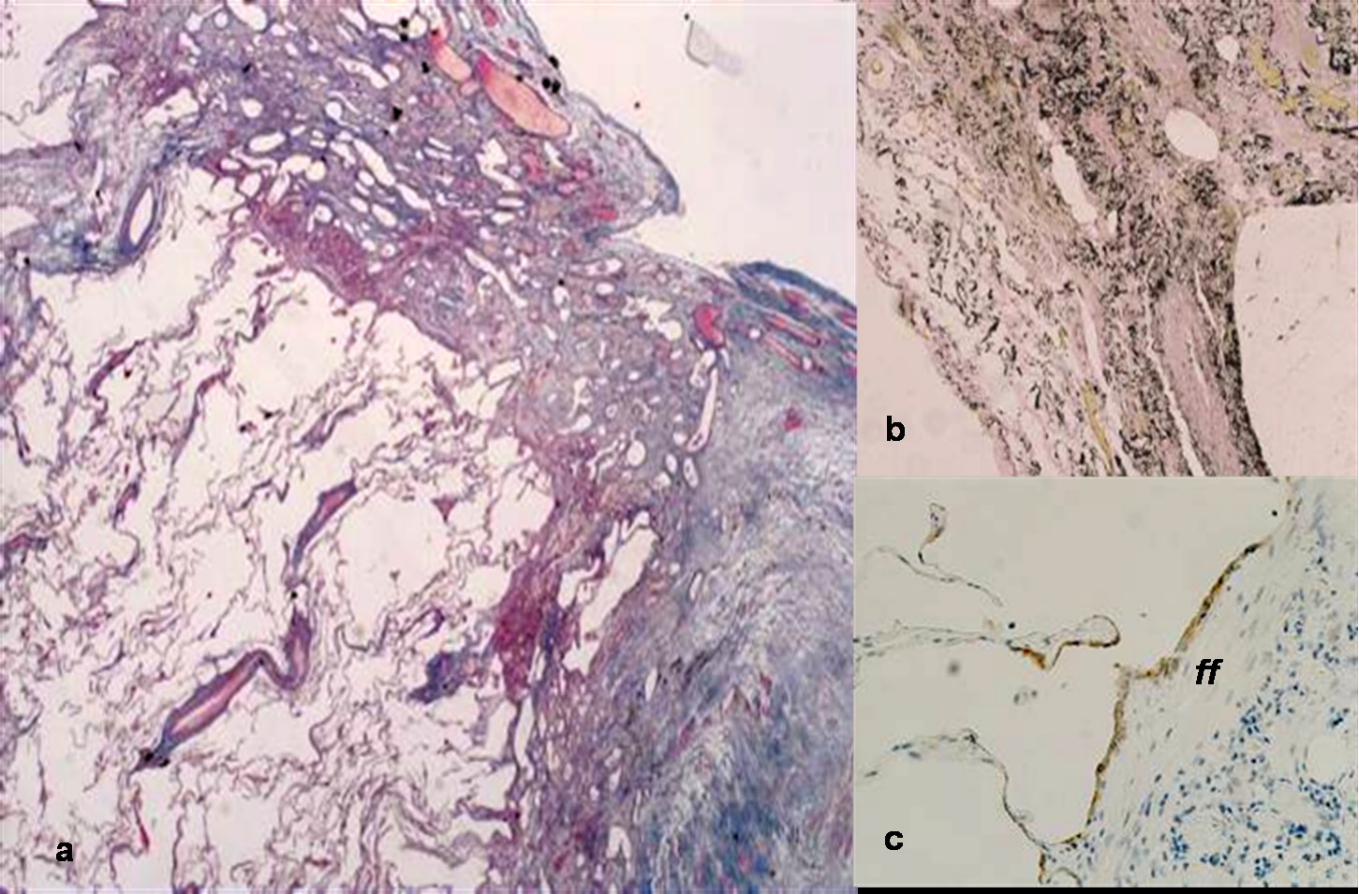
**Histological features of patient no. 2**. Marked subpleural thickening can be observed with sparing of adjacent lung parenchyma (4A). Fibroblast focus (ff) covered by alveolar epithelial cells is highlighted at the boundary between fibroelastic (4B, elastic stain) tissue and alveolar parenchyma (4C, cytokeratin 8/18 immunohistochemistry).

In the second case, a 28-year-old male, non-smoker, plumber, with suspected occupational exposure to asbestos, presented in 2004 with cough, vomiting and muscular asthenia. Serological exams showed positive precipitins for Aspergillus and birds. Functional respiratory test showed important signs of restrictive disease associated with a severe substantial reduction in DLCO. Chest X rays revealed interstitial prominence in apical lung zones and pleural thickening. High resolution CT scan (Figure 3) showed some calcified plaques, without upper or  lower lobe predominance. Significant fibrotic thickening of marginal pleura and of fissures was also seen in both upper lobes. Moreover, a reticular pattern associated with mild interlobular septal thickening, especially in apical subpleural regions, was observed.No honeycombing or pleural effusion was seen. Even though pleural plaques were likely related to asbestos exposure, pneumoconiosis did not justify all radiological findings, especially the fibrotic thickening of the subpleural region in the upper lobes. For this reason, patient underwent a transbronchial biopsy which did not reveal any  ferruginous bodies. An open lung biopsy was subsequently performed on the dorsal  segment of the right upper and lower lobes and it showed histological features of IPPFE (Figure 4).  

In both cases described in this letter, the histology corresponded to that previously described for IPPFE [1,2]. Visceral pleura was diffusely and markedly thickened by a mixture of elastic and dense collagen fibers, with sparing of adjacent lung parenchyma (Figure 2 and 4). Although the transition from fibroelastosis to normal parenchyma was abrupt, elastic fibers variably extended to adjacent alveolar walls. Some fibroblast foci were observed in both cases covered by alveolar epithelium at the boundary between the fibroelastosis and normal parenchyma. Scattered alveolar structures were covered by cuboidal type-II pneumocytes, as defined by the immunohistochemical expression of cytokeratin 8/18 and surfactant-protein-A. These alveolar structures were also entrapped within the fibroelastotic subpleural tissue, together with numerous vessels, including podoplanin-expressing lymphatic vessels (Figure 2A and 4B).

**Figure 5:**
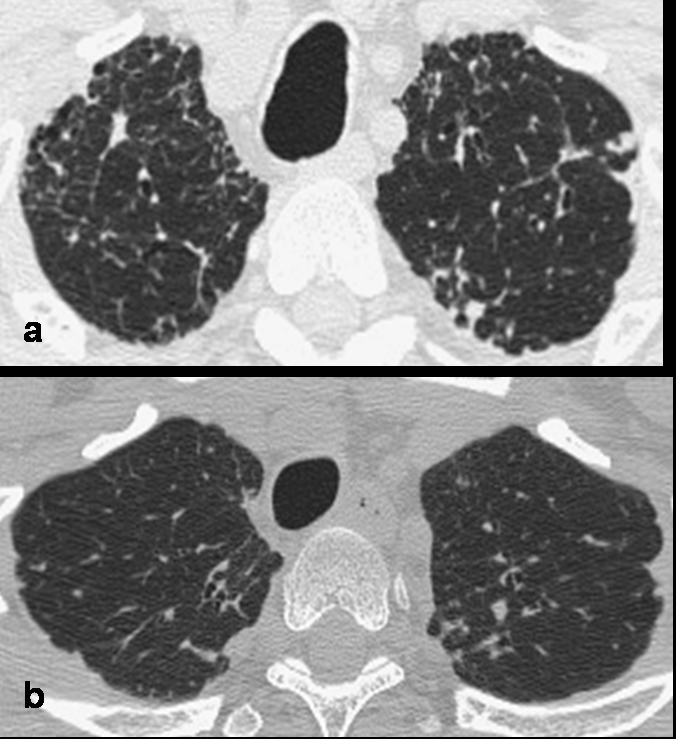
**CT scan of apical region in both patients (a: patient n°1 and b: patient n° 2) shows a diffuse thickening of pleuroparenchymal interface, suggesting IPPFE**.

Idiopathic pleuroparenchymal fibroelastosis (IPPFE) is a recently described entity characterized by marked pleural and sub pleural fibrosis, with main presentation typically in the upper lobes. In this letter, the first patient described showed honeycombing in the upper lobes, which is not suggestive of UIP. We considered the possibility of HP because of the prevalence of fibrotic changes in the upper lobes and of the mild exposure of the patient to countryside antigens. However, in a retrospective revision as the predominant pattern was thickening of pleural and subpleural parenchyma, HP was ruled out. The second patient's exposure history initially pointed towards asbestosis, but transbronchial and open lung biopsies did not reveal any ferruginous bodies, and the repeated radiology showed stability over five years (from 2004 to 2008). According to literature and to our observation, IPPFE could be considered to express two aspects of the same entity: a sporadic form, prevalent in male patients and a familiar form, mainly in young women in whom the disease is more aggressive and the prognosis is poor [1-4]. On the basis of this distinction and on the stability of follow-up, the second patient showed features of the sporadic form (Figure 5).

The differential diagnosis of IPPFE includes: asbestosis, connective tissue diseases, advanced fibrosing sarcoidosis and radiation or drug induced lung disease. Radiological and histological features of IPPFE may overlap with that disorders that mainly involve the upper lobes: chronic hypersensitivity pneumonitis and apical caps. In hypersensitivity Pneumonitis (HP) lesions are bronchiolocentric and bronchiolization of the centrilobular airways is prominent. Apical caps may be difficult  to distinguish from IPPFE and probably the difference in mainly quantitative. Differential diagnosis could be often reached on the basis of clinical and radiological features: IPPFE is an interstitial lung disease in opposite to apical cap [2,3]. In the histological setting, a last differential diagnosis may be done with findings observed in emphysematous scars at the apex of lung. Also, in smoking-related disease, a ialine fibrosis surrounding subpleural and centrilobular emphysema can be seen: it could make easier the differential diagnosis. It needs to be underlined, that probably IPPFE is, as far as we know, a not specific morphological reaction that might be associated also to different causes or settings. The possibility of IPPFE should be considered when radiological evidence is not consistent with well-defined idiopathic pneumonias that affect upper lobes. 

## Competing interests

The authors declare that they have no competing interests.

## Consent

Written informed consent was obtained from the patient for publication of this case report and accompanying images. A copy of the written consent is available for review by the Editor-in-Chief of this journal.
